# TiO_2_-based Photocatalytic Cementitious Composites: Materials, Properties, Influential Parameters, and Assessment Techniques

**DOI:** 10.3390/nano9101444

**Published:** 2019-10-11

**Authors:** Fatemeh Hamidi, Farhad Aslani

**Affiliations:** 1 Materials and Structures Innovation Group, School of Engineering, University of Western Australia, Crawley 6009, WA, Australia; Fatemeh.Hamidi.Technosa@outlook.com; 2 School of Engineering, Edith Cowan University, Joondalup 6027, WA, Australia

**Keywords:** self-cleaning surfaces, air purification, antimicrobial surfaces, TiO_2_, photocatalysis cementitious materials

## Abstract

Applications of heterogeneous photocatalytic processes based on semiconductor particles in cement-based materials have received great attention in recent years to enhance the aesthetic durability of buildings and reducing global environmental pollution. Amongst all, titanium dioxide (TiO_2_) is the most widely used semiconductor particle in structural materials with photocatalytic activity because of its low cost, chemically stable nature, and absence of toxicity. Utilization of TiO_2_ in combination with cement-based materials would plunge the concentration of urban pollutants such as NO_x_. In fact, cementitious composites containing TiO_2_ have already found applications in self-cleaning buildings, antimicrobial surfaces, and air-purifying structures. This paper aims to present a comprehensive review on TiO_2_-based photocatalysis cement technology, its practical applications, and research gaps for further progression of cementitious materials with photocatalytic activity.

## 1. Introduction

**Figure 1:**
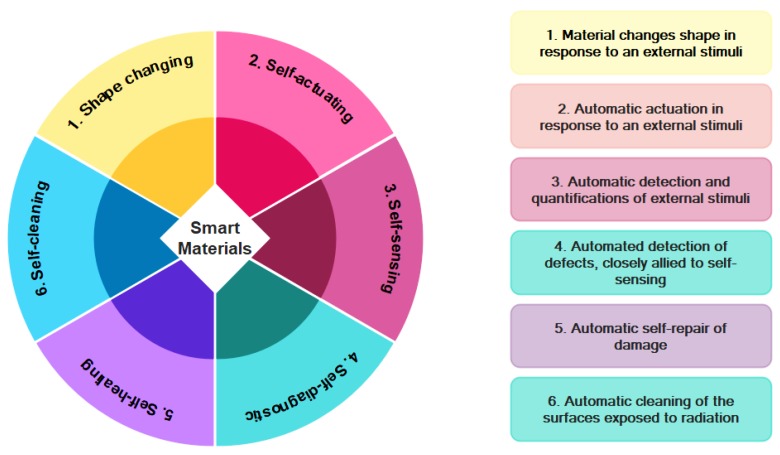
Smart functions added to the structural materials and their capabilities.

Cement-based materials/composites have been utilized in civil engineering structures for many centuries and remain the dominant materials in the construction industry. However, in their modern applications, not only they are applied as structural materials, but they are also used as functional materials to design and fabricate smart structures [1,2]. Smart materials are engineered materials capable of representing a unique beneficial response to an external stimuli [3]. Figure 1 shows some examples of the smart materials and their capabilities. For instance, self-cleaning mortar/concrete is capable of maintaining the aesthetic characteristics of buildings, such as color, over time even in harsh urban environments [4,5]. In this regard, nanotechnology is of great importance towards construction of functional buildings. By addition of nanosized materials to the traditional structural materials, it would be possible to not only promote the basic properties of cementitious materials but also add certain functionality to them, including self-cleaning, antimicrobial, and pollution-reducing properties. On the other hand, the enormous resource of energy from sunlight and the urgent demand for a cleaner environment have rendered architects and structural engineers to redesign structures in order to utilize sunlight in combination with functional engineered structural materials to lower energy usage and environmental pollutants. By being benefited from the privileges of nanotechnology, improved cementitious materials in terms of durability and strength are achievable, thereby increasing the quality and longevity of structures as well as lowering the costs of renewing civil infrastructures [6].

After discovering the photocatalytic splitting of water in a titanium oxide (TiO_2_) anode photochemical cell by Fujishima and Honda [7] as well as Wrighton et al. [8] in 1970s, the fundamentals and applications of photocatalysis have received global attention [9], due to the promising applications of heterogeneous photocatalysis, in various fields including solar energy, green chemistry, and environmental remediation [10,11,12,13,14]. It has now been over one decade since photocatalysis was first utilized in different materials, particularly cement binders, to achieve self-cleaning and, more recently, depolluting effects [5,7,11]. Surging air pollutants in urban regions has led researchers to utilize photocatalytic properties in order to eliminate the substances contaminating the atmosphere. In fact, photocatalysis efficiently contributes to enhancing quality of life.

TiO_2_, particularly nanosized TiO_2_ [6], is the most widely used component in photocatalysis structural materials because of its compatibility with conventional building materials, such as cement, without deteriorating their performances. It is a semiconductor material that, in traditional applications, has been used as white pigment [4,11]. It has been reported by various researchers that TiO_2_ is effectively able to reduce pollutants such as nitrogen oxides (NO_x_), aromatics, ammonia, and aldehydes [15,16,17]. Regarding construction materials, TiO_2_ is usually incorporated in the concrete bulk; however, it can be applied to the surface of building materials as coating [4,5,18]. The implementation of photocatalysis materials in combination with structural materials started in the early 1990s, and photocatalytic paving blocks and coatings based on hydraulic binders have already been patented by Mitsubishi Materials Corporation and Italcementi S.p.A [18,19,20,21,22,23]. The numerous functions of TiO_2_ as a structural and functional material has led to its wide applications in both interior and exterior structural materials, such as cement mortars, paving blocks, and exterior ties, in order to construct smart functional buildings that have self-cleaning and antimicrobial properties and, more importantly, help to clean the air and environment [4].

This paper represents a comprehensive review of TiO_2_-based photocatalysis cementitious materials, establishing the concepts of self-cleaning buildings, antimicrobial surfaces, and depolluting effects. Moreover, the properties of photocatalysis cement-based materials in fresh and hardened states, the influential parameters on the photocatalytic activity, as well as the testing methods to assess the efficiency of the photocatalysis process will be reviewed. The potential applications of photocatalysis structural materials and their future directions will be addressed as well.

## 2. Heterogeneous Photocatalysis Process

A photocatalyst is a compound that facilitates a chemical reaction upon absorption of light and is generated in the process [18,24]. Many transition metal oxides show photocatalytic activity, that is, these substances would act as a photocatalyst and promote oxidation and reduction reactions when exposed to electromagnetic radiation [21].

**Figure 2:**
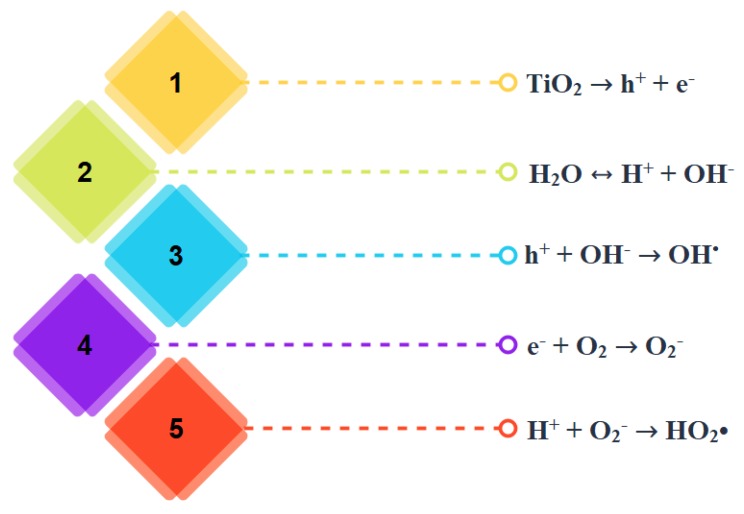
Photocatalytic reactions based on the electronic structure of semiconductors.

The basic heterogeneous photocatalytic process is the activation of a semiconductor photocatalyst by irradiation [25]. TiO_2_, ZnO, and CdS are widely used semiconductor materials [26]. Semiconductors contain a filled conduction band and an empty valence band, which are separated by a band gap of energy (E_g_). By absorbing a proton of energy equal to or larger than E_g_, an electron (e^−^) from the valence band would be promoted to the conduction band resulting in a hole (h^+^) in the valence band. The valence band hole is a strong oxidizing agent and is capable of oxidizing electron donor molecules adsorbed on the surface, whereas the conduction band electron is a powerful reducing agent and would reduce acceptor molecules [16,24]. Figure 2 represents the sequential photocatalytic reactions based on the electronic structure of semiconductors. Reactive oxygen species have the capability to decompose microbes to CO_2_ and H_2_O [27,28,29].

The efficiency of the photochemical process is a complex function of several factors, in which five of them are the most influential: (1) effective absorption of sunlight, (2) quick charge separation after light absorption to prevent electron-hole recombination, (3) product separation from the photocatalyst’s surface, (4) compatibility between the redox potentials of the valance band hole and conduction band electron with those of the donor and acceptor species, respectively, and (5) long-term stability of the photocatalyst [6,17,22,24,25].

Among all the transition metal oxides, TiO_2_ is the most studied photocatalyst for self-cleaning cementitious materials because of its low cost, chemical stability and human safety, non-toxicity, and efficient photocatalytic activity [11,26,30]. Under ambient conditions, TiO_2_ has three main crystal structures including anatase (distorted tetragonal crystal structure), rutile (also tetragonal), and brookite (orthorhombic crystal structure), in which only rutile and anatase are attractive for practical applications since they are wide band gap semiconductors [17,21,25,31]. In general, anatase is more efficient in degrading both organic and inorganic pollutants in vapor and/or liquid phases [31,32]. Rutile and brookite phases are more applicable for the selective oxidation of organic syntheses [15,16,33]. However, coupling of anatase and rutile phases would increase the photocatalytic activity significantly compared to each individual component [34].

**Figure 3:**
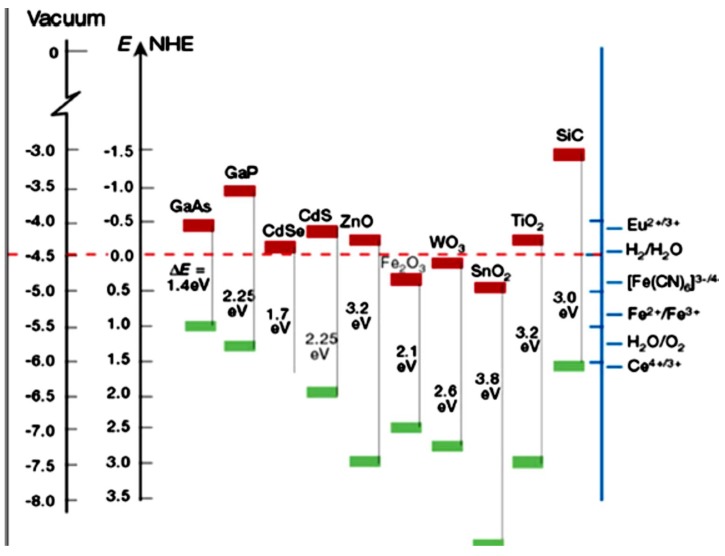
The band gap positions for various photocatalysts (E indicates the electric potential, and NHE stands for normal hydrogen electrode potential) [39].

**Figure 4:**
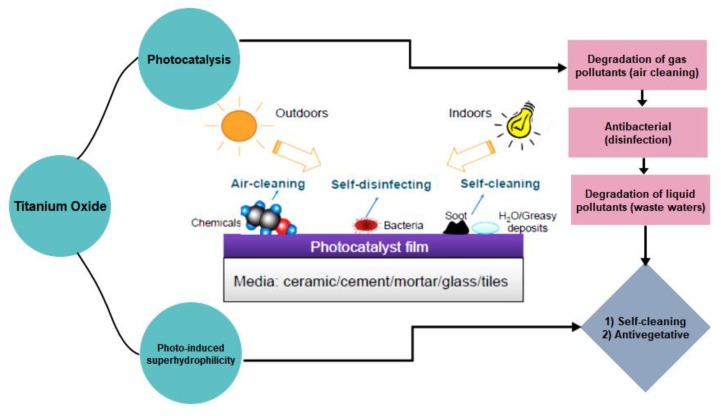
Main applications of the photocatalytic activity of TiO_2_ as appearing in the literature [4]. Photo from [40].

The band gap of anatase is on the order of 3.2 eV (Figure 3), corresponding to a wavelength of 388 nm, meaning that its activation needs an irradiation source with wavelength lower than 388 nm, which is in the near-UV region. Therefore, visible light is not sufficiently energetic to induce photocatalytic activity in anatase [18,21,24]. The high photocatalytic activity of anatase has led to its extensive applications as photocatalytic coatings on various substrates under low-intensity, near-UV light [11,35,36]. Figure 4 summarizes the main photocatalytic applications of TiO_2_ as reported in the relevant literature.

Regarding the influential parameters, numerous physico-chemical variables have impacts on the photocatalytic properties of TiO_2_, namely particle size, surface area, pore volume, surface hydroxyl content, and crystallinity degree [15,25,37]. Crystallinity, in particular, is an important factor contributing to the high photoactivity since the presence of an amorphous phase would facilitate the recombination of photo-excited electrons and holes [17,32,37,38].

## 3. Photocatalysis Cementitious Materials

**Figure 5:**
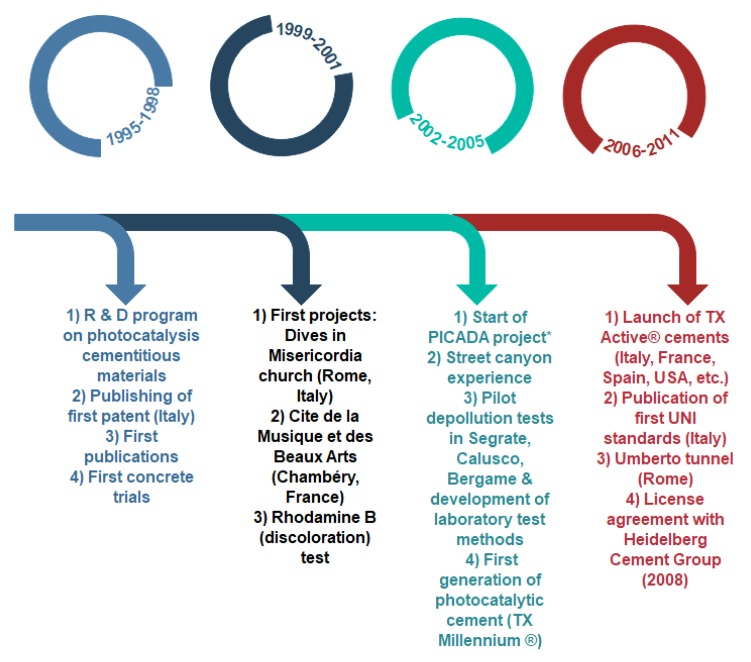
History of photocatalysis cementitious materials [6,41,42]. (Note: PICADA project: European project as Photocatalytic Innovative Coverings Applications for Depollution Assessment.)

Photocatalysis cementitious materials have been studied as an alternative to eliminate environmental pollution through the use of construction materials containing photocatalyst compounds. Moreover, maintaining the aesthetic characteristics of structures, especially those based on white cement, was another important pillar to develop photocatalysis cement-based materials [5,18,24]. Figure 5 summarizes the history of photocatalysis cement-based materials. Because of the addition of TiO_2_ in the bulk of the structure, it is expected that the concrete technology and the final properties of the cementitious products would not be affected, meaning that both white and grey cement can be used without any particular problems [5]. However, organic admixtures and other supplementary cementitious materials must be selected carefully to not interfere with the photocatalytic activity of the products [31].

**Figure 6:**
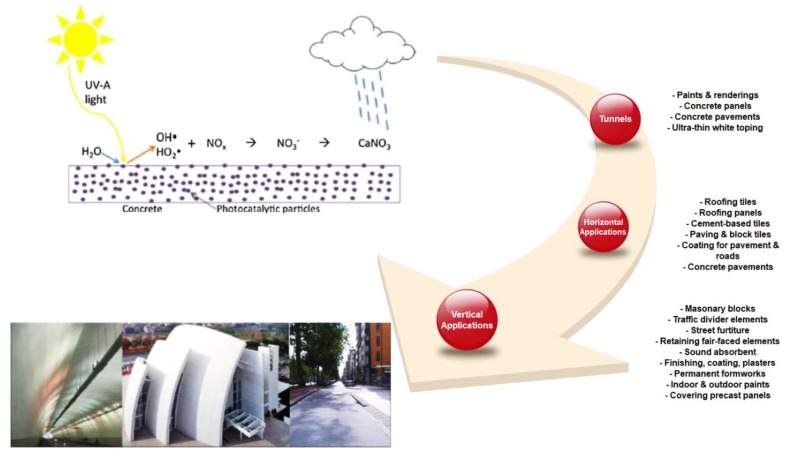
Possible applications of photocatalysis cement-based materials as reported in the literature (schematic of the photocatalytic concrete from [6]). From left to right: painting of Umberto tunnel in Rome, Dives in Misericordia church in Rome [41], and pavement blocks on Leien of Antwerp in Belgium [18].

Utilizing photocatalytic cementitious materials, several buildings have been designed and constructed since 2000 including a church, Dives in Misericordia, in Italy; music and arts city hall, Chambéry, in France; police central station, Bordeaux, in France; air France building, Roissy-Charles de Gaulle Airport, in France; and Saint John’s court in Monaco [22,24,43]. The most common TiO_2_ applications in cementitious materials are categorized as vertical, horizontal, and tunnel applications. Figure 6 represents examples of the practical applications of TiO_2_-based cementitious materials.

### 3.1. Self-Cleaning Surfaces

**Figure 7:**
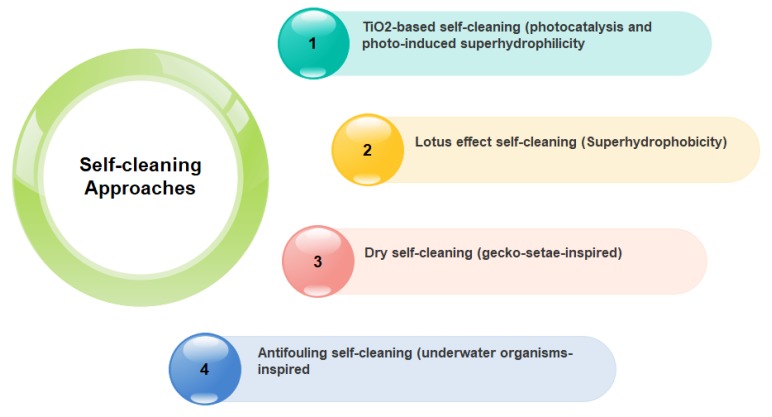
Self-cleaning approaches in materials.

Self-cleaning is a favorable property in terms of contamination-free surfaces [44,45,46,47,48]. So far, many different synthesis strategies have been developed to design and fabricate self-cleaning surfaces [49,50,51,52,53]. Such surfaces are able to reduce the costs associated with maintaining the clean appearance of a range of surfaces in civil infrastructures [6,43]. A great variety of these surfaces for numerous applications have been commercialized. Regarding the mechanism of self-cleaning, these materials fall into four main categories [49,54], represented in Figure 7.

**Figure 8:**
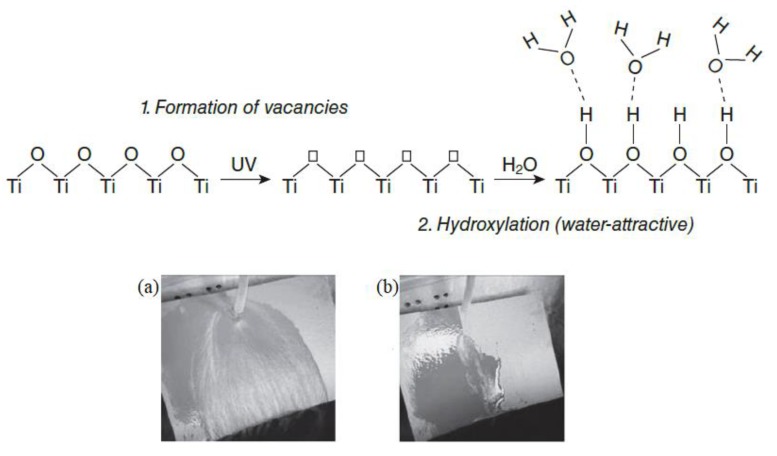
An illustration of the possible photo-induced superhydrophilicity mechanism of TiO_2_ and its practical application. The photograph represents a plastic sheet (**a**) covered with TiO_2_, representing the superhydrophilic property, and (**b**) without TiO_2_ coating, representing hydrophobic behavior [21].

Superhydrophilicity is an important property to achieve self-cleaning functions in materials. Water droplets can spread out, generating a thin film on superhydrophilic surfaces. By spraying water onto such surfaces by means of rain or light, water can diffuse into the space between the substrate and the dust, eliminating the dust [55]. Hydrophilic surfaces have a water contact angle of less than 90°, and, in the case of superhydrophilic surfaces, the water contact angle is close to 0° [56]. Materials with photocatalytic activities are the most common substances for hydrophilic surfaces. Figure 8 represents the possible photocatalytic superhydrophilicity mechanism of TiO_2_ [55].

Among the numerous materials with superhydrophilic properties, TiO_2_ is one of the most promising because of its favorable physical and chemical properties. TiO_2_ can exhibit both photocatalytic and photo-induced superhydrophilicity properties. TiO_2_ has been extensively investigated over the past decade; however, further research is being carried out to identify the exact mechanisms for the destruction of specific pollutants. Nevertheless, it is difficult to distinguish whether photocatalysis or photo-induced superhydrophilicity is more important for self-cleaning properties [4,57].

Superhydrophilicity is of great importance in civil structures since it would prolong the aesthetic durability of the structures. Hydrophilic surfaces with photocatalytic activities are further advantageous because of their capability to decompose a broad range of organic pollutants, such as aromatics, surfactants, and dyes [43,58], as well as many compounds available in the stains on the outdoor surfaces in the presence of oxygen [59].

Providing more hydroxyl radicals on the surface of TiO_2_ through superhydrophilicity would lead to higher efficiency of degradation of organic substances [60]. Moreover, the transition between hydrophilicity to hydrophobicity is possible due to the adsorption of organic compounds on the film surface; therefore, a more efficient photocatalytic decomposition of these organic contaminants would result in maintaining the superhydrophilicity of the surface [61]. Thus, the simultaneous effects of photocatalysis and superhydrophilicity will assure that the self-cleaning behavior of TiO_2_ films would be preserved [62].

### 3.2. Antimicrobial Surfaces

**Figure 9:**
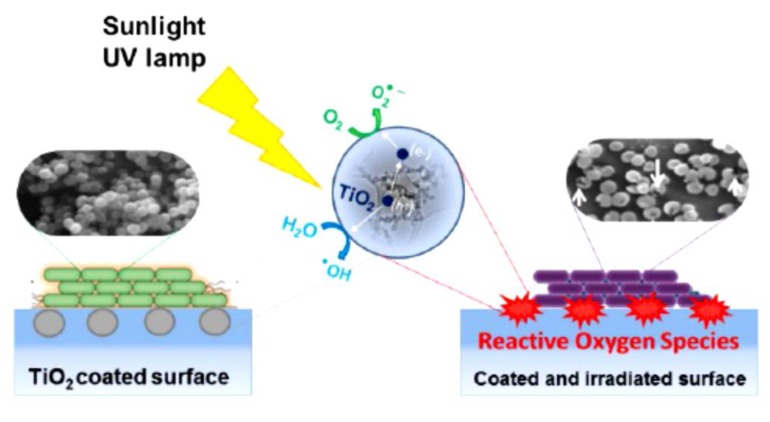
Schematic of self-disinfecting surfaces [67].

Growing concerns for human health and quality of life have led to the implementation of nanoparticle photocatalysts in civil structures to fabricate self-disinfecting surfaces, mostly for public places that need a high level of hygiene, such as in hospitals, schools, public transportation, and so on [4,6,63]. The self-disinfecting property of semiconductor particles such as TiO_2_ and ZnO mainly is due to their photocatalytic activities. Figure 9 illustrates the photocatalytic process occurring on the self-disinfecting surfaces. In ceramic and building industries, there is a growing interest for the photo-induced antimicrobial effect of TiO_2_, particularly for microbiologically sensitive environments such as medical facilities. As relevant studies reveal [11,36], installing photocatalytic tiles in indoor furnishing not only reduced the amount of bacteria on the wall surface to a negligible level, but it also decreased the amount of bacteria in the air significantly. Moreover, the photo-induced antimicrobial activity of TiO_2_ can also be applied to control biological growth on the concrete surface [4]. Growth of biofilm on concrete surfaces would lead to the loss of aesthetic appearance of the buildings and deteriorate the durability of concrete structures [64,65]. As reported by Linkous et al. [66], coated cement substrate with a dispersion of 10 wt% TiO_2_ powder would decrease the algae growth by 66% as compared to an unprotected cement surface. According to them, by addition of 1.0 wt% of a noble metal, such as Pt or Ir, to the photocatalyst, an 87% reduction in algae growth was observed.

The effectiveness of antimicrobial nanoparticle photocatalysts is limited to the environment, meaning that there must be sufficient irradiation with a required wavelength (388 nm UV light for TiO_2_). As an alternative, doping of TiO_2_ to decrease its band gap would result in the activation of the photocatalytic process by visible light, which will promote indoor photocatalytic activity. It has been reported that doping of TiO_2_ with noble metals (i.e., Ag, Ni, Pt, Au, Cu, Rh, Pd), oxides (i.e., ZnO, WO_3_, SiO_3_, CrO_3_), or nonmetals (i.e., C, N, S, P) would be effective [6]. However, doping of nano-TiO_2_ with noble metals is expensive [68]. Thus, substituting TiO_2_ with other photocatalyst nanoparticles with better antimicrobial activities, such as ZnO [69], could be considered as a viable alternative.

### 3.3. Air-Purifying Surfaces

Air pollution due to nitrogen oxides (NO_x_) is a dramatic issue that contributes to exacerbating quality of life, especially in large urban areas [70,71,72]. NO_x_ together with sulfur oxides (SO_x_) are the main chemical compounds responsible for acid rain and photochemical smog [22,73]. Indoor air pollution from substances including NO_x_, which, in atmospheric chemistry, is the sum of nitric oxide (NO) and nitrogen dioxide (NO_2_), carbon oxides (i.e., CO and CO_2_), and volatile organic compounds (VOCs), not only threatens human health but also seriously affects plant regular metabolism [73]. Amongst all, NO_x_ are the most problematic pollutants. NO is considered the primary pollutant, which is mainly introduced into the atmosphere directly from high-temperature combustion in transport and industrial activities, whereas NO_2_ is considered as a secondary pollutant since it is mostly formed in the atmosphere due to the interaction between NO with O_2_ or O_3,_ and/or sunlight [73,74].

**Figure 10:**
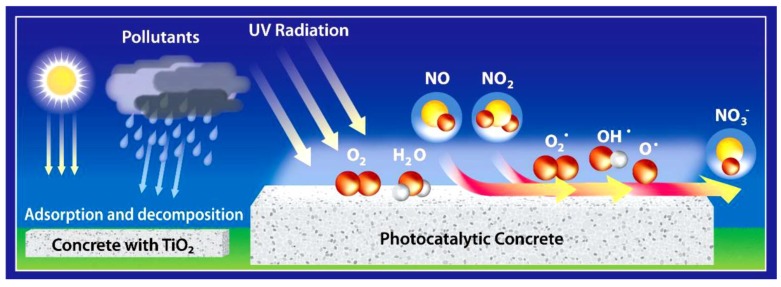
Schematic illustration of NO_x_ removal by photocatalysis concrete pavement [94].

Photocatalysts are capable of decomposing various numbers of oxides and organic compound pollutants that cause health and environmental problems. The governing decomposition mechanism involves the generation of radicals due to the irradiation to the photocatalyst substance, and subsequently converting pollutants into harmless compounds [40,75,76]. The accepted reaction mechanism for the photocatalytic conversion of NO_x_ compounds is represented in Equations (1)–(3) [4,42,77,78]. NO_3_^−^ is harmless in small quantities and would be washed away by water droplets [77,79]. Figure 10 shows the photocatalytic reaction to eliminate NO_x_ pollutants by photocatalysis concrete pavements.

NO + HO_2_• → NO_2_ + OH•; (1)

NO + OH• → NO_2_ + H^+^; (2)

NO_2_ + OH• → NO_3_^-^ + H^+^. (3)

Similarly, purification of SO_2_ is as follows [31,42,80]:

SO_2_ + OH• → HSO_3_; (4)

HSO_3_ + SO_2_ → SO_3_; (5)

SO_3_ + H_2_O → H_2_SO_4_; (6)

HSO_3_ + HO• → H_2_SO_4_. (7)

**Figure 11:**
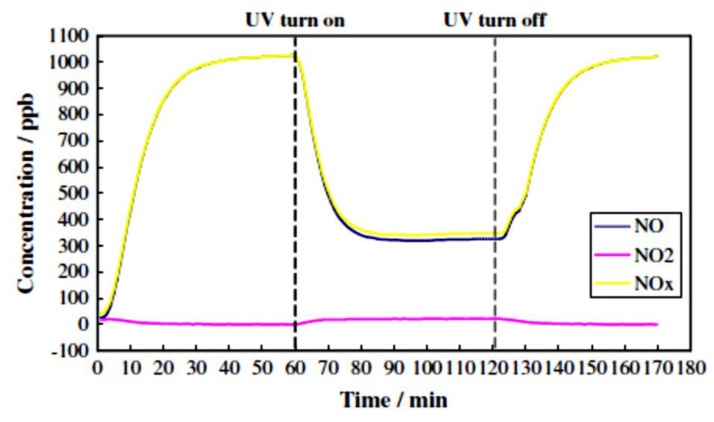
NO_x_ removal pattern by photocatalytic pavement blocks obtained from laboratory test methods [4].

The first report on photocatalytic decomposition of pollutants based on TiO_2_ was published in 1977, in which the capability of the photocatalytic process to degrade cyanide into a harmless product in wastewater was reported [81]. In recent years, the removal of organic and inorganic contaminants in air through the photocatalytic process has been explored extensively owing to the potential of photocatalysts to purify air in offices, buildings, homes, schools, and so on [82]. Amongst the most used technologies for NO_x_ remediation [83,84], photocatalytic degradation of NO_x_ has become a valid alternative in recent decades, confirmed by tremendous scientific attempts [78,82,85,86,87,88,89,90,91,92,93] and the continuous growth of commercial products available in the market, which are mainly cements and paints containing TiO_2_ [19,43]. According to laboratory evaluations, a typical pattern of NO_x_ removal by the photocatalytic paving blocks is represented in Figure 11. Photocatalysts, such as TiO_2_, can be easily implemented in cementitious materials and paints, thereby creating air-purifying surfaces in a broad range of structures and infrastructures including pavement blocks [42,94,95], filters and membranes for indoor/outdoor air purification, and so on [72,75].

Concrete pavement and external building surfaces are ideal for incorporating photocatalytic materials since their flat configurations would ease the exposure of the photocatalyst to sunlight [95]. In photocatalytic cements, the formed NO_3_^−^ reacts with calcium in the cement to form a water-soluble salt, calcium nitrate, which can be removed by rainwater easily. Efficient elimination of air pollutants with concentrations in the range of 0.1–10 ppm is possible by means of such photocatalytic cementitious materials [57]. Many laboratory studies have been conducted to demonstrate the depolluting effect of photocatalytic cement-based materials for eliminating VOCs, NO_x_, CO, toluene, lead, and SO_2_, which could be found in detail in [77,78,80,93,96,97,98,99,100,101,102,103,104,105]. Moreover, Etxeberria et al. [106] explored the impact of dust and oil accumulation on the efficiency of concrete surfaces with photocatalytic activity in removal of NO_x_. They reported that dust accumulation would result in partial loss of efficiency in removing NO_x_ for TiO_2_-coated concrete, while for the samples in which TiO_2_ was included in the concrete, severe loss of efficiency was observed. Oil impregnation also led to the complete loss of photocatalytic efficiency for concrete containing TiO_2_, whereas for TiO_2_-coated concrete, the initial NO_x_ removal capacity showed 80%–90% decrease because the TiO_2_-coated concrete had better accessibility to UV light.

## 4. Properties of Photocatalysis Cementitious Materials

### 4.1. Microstructure

**Figure 12:**
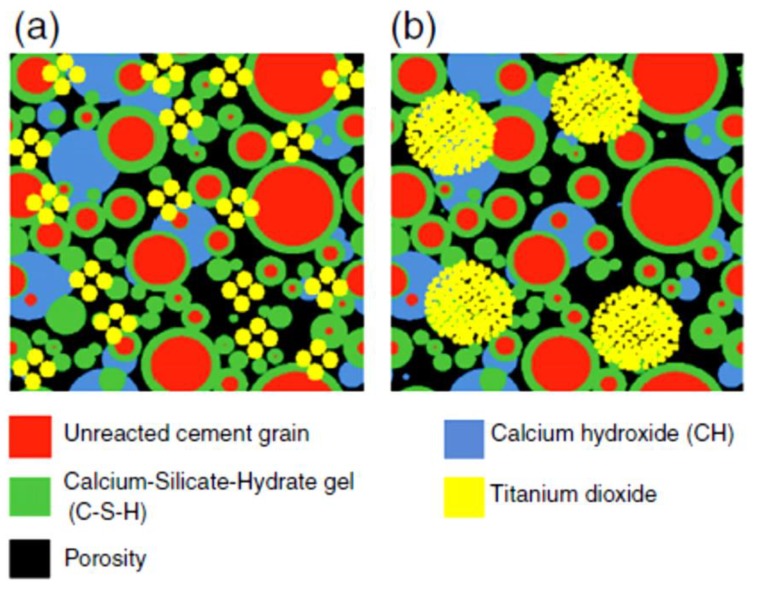
Hardened cement structures containing (**a**) micro- and (**b**) nano-sized TiO_2_ [113].

The mechanical properties of TiO_2_-based cementitious materials strongly rely on the hydration products and microstructure of cement composites [31]. It has been demonstrated that in particular circumstances—including high pH, the presence of non-indifferent electrolytes such as Ca^2+^, and high ionic activity, which are typical conditions within the cement paste—both nano- and microsized TiO_2_ particles represent great tendency towards agglomeration because of the ion–ion correlation phenomena [107,108,109,110] that are similar to calcium silicate hydrate (C-S-H) particles in cement [111,112]. However, the structure of particle clusters containing C-S-H gel is completely different from those of TiO_2_ agglomerate [107]. Comparing nano- and microsized titania particles, microsized TiO_2_ aggregates are smaller and have larger pores and a better dispersion than nano-TiO_2_ [113]. Chemical surface treatments of micro- and nano-TiO_2_ with phosphorous and potassium, aimed at enhancing and controlling the crystal growth and facilitating dispersion in aqueous systems, carried out by Folli et al. [107], revealed small, deflocculated, and highly dispersed agglomerates for micro-TiO_2_, whereas for nano-TiO_2_, large, flocculated agglomerates with weak dispersions were observed. In Figure 12, the models of TiO_2_-based cement composites are represented showing the agglomeration/dispersion features.

According to such experimental evidence, the photocatalytic activity of cementitious materials is a function of the accessible surface area in the hardened cement structure rather than the specific surface area of nano-TiO_2_ [114,115]. For instance, large molecules, such as rhodamine B (RhB) with an average molecule diameter of 1.6 nm [116], can penetrate into the nano-TiO_2_ clusters (pore size around 8 nm) with difficulty, whereas it would be easy to access micro-TiO_2_ clusters [107,113]. The smaller and well-dispersed micro-TiO_2_ clusters on the surface of the cement specimens, together with their macropores, could be more efficient than larger and poorly dispersed nano-TiO_2_ clusters in terms of offering a larger accessible surface area for adsorption and, subsequently, reaction of large molecules such as rhodamine B. Notwithstanding, smaller molecules, such as gaseous NO_x_ with dimensions of 100–200 pm, can penetrate easily into both nano- and micro-TiO_2_ clusters and have access to a higher surface area in both catalysts [113]. In such circumstances, the higher specific surface area of nanosized TiO_2_ is an indicator for its high photocatalytic capability for NO_x_ degradation.

**Figure 13:**
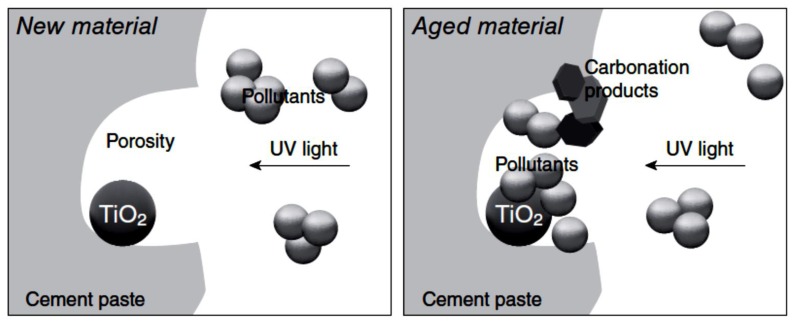
Effect of aging on the possible shielding effect of photocatalytic cementitious materials [21].

The alkaline environment of both hydraulic binders (i.e., cement and hydraulic lime) and nonhydraulic binders (i.e., gypsum and lime) does affect the photocatalytic activity of TiO_2_. These binders are typically porous at the micro- and nanoscale. These pores are where TiO_2_ usually would be placed, acting as a further aggregate or nanofiller. The unbounded hydration products in the material porosities would be able to be adsorbed on the TiO_2_ surface and, therefore, reduce the available surface area of the photocatalyst. Moreover, an increase in electron-hole recombination could also occur on adsorbed species [117,118]. Furthermore, by material aging, the alkaline materials would be carbonated and, therefore, induce a solid volume increase of higher than 10%, resulting in a decline in capillary absorption and precipitation of calcium carbonate [119,120]. These precipitates would intercept the active sites of the photocatalyst and decrease the photocatalytic efficiency of TiO_2_, for shielding effects in particular [4]. The situation would be exacerbated by the accumulation of contaminants on the surfaces exposed to the environment [21]. Hence, preserving the long-term efficiency of the photocatalytic activity within an alkaline environment of cementitious materials would be challenging. Figure 13 represents the shielding effect in young and aged cement matrixes.

Now, the question arises on how the incorporated photocatalyst would affect the microstructure of the binder. Lackhoff et al. [117] and Li et al. [121] emphasized that TiO_2_ has pozzolanic activity, supported by an observed acceleration in cement hydration by Li et al. [121], while Lackhoff et al. [117] had no validating data. By a reduction in the setting time and final porosity of TiO_2_-based photocatalytic cementitious materials, Nazari and Riahi [122] supported the idea of the pozzolanic activity of TiO_2_, whereas Chen and Poon [4] rejected any pozzolanic activity of TiO_2_ since no mass change was observed for TiO_2_ during cement hydration, suggesting the inert behavior of the titania nanopowder. Thus, the pozzolanic activity of TiO_2_ still needs further pioneering work.

As mentioned before, TiO_2_ would affect the pore structure of the cement paste as well. Zhang et al. [123] revealed that by addition of 1, 3, and 5 wt.% nano-TiO_2_, the most probable pore diameters after 28 days corresponded to 84, 53, and 47 nm respectively, while the pore diameter of the cement paste was 103 nm before addition of nano-TiO_2_. Moreover, by increasing the nano-TiO_2_ content, the accumulative pore volume decreased. Other researchers [124,125,126,127,128] reported a decreasing trend in the total specific pore volume by increasing the nano-TiO_2_ content. Li et al. [129] demonstrated that nano-TiO_2_ would enhance the compactness of cementitious composites and reduce their porosity from 9.045% to 6.96%. Likewise, Salman et al. [130] observed that the nano-TiO_2_ is capable of filling the pores within the cement matrix, reducing the size of calcium hydrate crystals, and densifying the microstructure of the cementitious composites [126].

### 4.2. Fresh State Properties

#### 4.2.1. Hydration Process

As mentioned before, there are some contradictions related to the pozzolanic activity of TiO_2_ and the way it impacts on the hydration process of the cement matrix. Various researchers reported promotion in the hydration process of the cement paste, indicating the pozzolanic activity of TiO_2_ [125,127,129,130,131,132,133], while there are some reports on the inert nature of it [107,134]. According to Chen et al. [135] and Zhang et al. [123], an acceleration in cement hydration and an increase in the intensity of CH (calcium hydroxide) were observed, supporting the idea of TiO_2_ pozzolanic activity. Moreover, Jayapalan et al. [136] found that the incorporation of 10 wt.% nano-TiO_2_ with particle sizes of 15–25 nm in the cement matrix led to maximum promotion in the cement hydration process. Nevertheless, an agreement has not been reached yet on the reason of promoting the cement hydration by nano-TiO_2_. Recently, it has been claimed that nano-TiO_2_ is capable of enhancing the rate and the peak of cement hydration due to its nucleation and particle-filling effects without participation in the hydration process [137,138,139]. As Lawrence et al. [140] revealed, nano-TiO_2_ could act as a nucleus in the cement matrix to accelerate the formation of C-S-H gel.

Despite the evidence collected by researchers approving the positive impacts of nano-TiO_2_ on cement hydration, Kurihara et al. [132] published a report stating that implementation of nano-TiO_2_ in the cement matrix reduced the precipitation of CH, which led to a decrease in the cement hydration degree since the effect of nano-TiO_2_ on the cement matrix could vary regarding the type of cement matrix, water/cement ratio, nano-TiO_2_ content, nano-TiO_2_ size and type [115], and dispersion degree.

#### 4.2.2. Setting Time and Workability

Incorporation of nano-TiO_2_ would affect the setting time of the cement paste noticeably. Chen et al. [134], Soleymani et al. [127], and Zhang et al. [123] reported that, by inclusion of nano-TiO_2_, the initial setting time would be shortened since the incorporation of nano-TiO_2_, with a high specific surface area, in the cement matrix would increase the viscosity of the paste and, consequently, negatively impact the workability of cementitious composites containing nano-TiO_2_.

According to the relevant literature, the workability of the cement composites containing nano-TiO_2_ would decrease by increasing the dosage of nano-TiO_2_ [135,141,142,143], mostly due to the small size effect and high specific surface area. However, there are some reports which reveal that the addition of nano-TiO_2_ would deteriorate the workability of the cementitious composites only in typical conditions, meaning that under some special circumstances, nano-TiO_2_ would not impact the workability of the cement negatively [128,144,145,146]. For instance, applying nano-TiO_2_ to black rice husk ash mortars increased the fluidity of the mortars [144], while for nonhydraulic binders, such as lime stone, there was no evident change [128]. For lightweight, natural hydraulic lime (NHL)-based mortar, Giosuè et al. [147] observed a deteriorating impact of TiO_2_ addition on the workability of NHL-based mortar with expanded glass and expanded silicate since in the presence of fine TiO_2_ particles, more water would be required to wet the surface, as well as particles tend to agglomerate.

### 4.3. Hardened State Properties

#### 4.3.1. Compressive Strength

**Figure 14:**
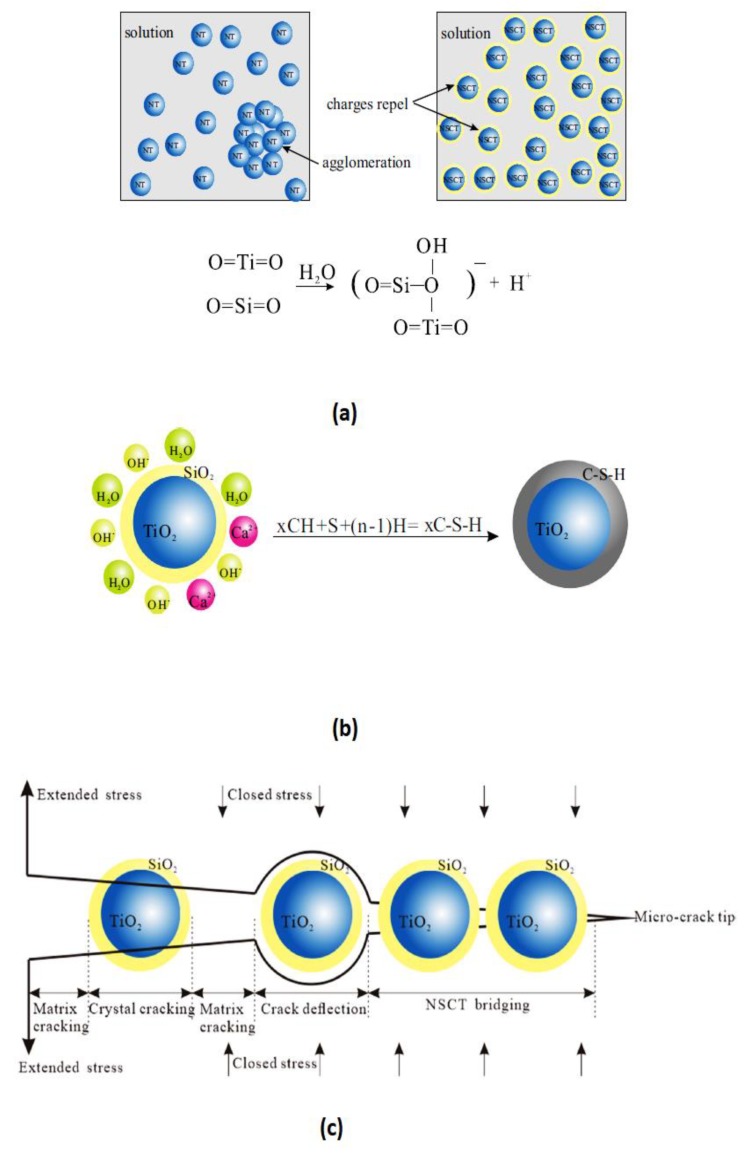
An illustration of SiO_2_-coated TiO_2_ nanoparticles on cementitious composites. (**a**) Improved dispersion due to repelling charges. (**b**) Pazzolanic activity of SiO_2_-coated TiO_2_. (**c**) Nanocore effect to control microcrack propagation [149]. (Note: NSCT is Nano SiO_2_-coated TiO_2_).

As reported by various researchers, the addition of nano-TiO_2_ would enhance the compressive strength of the cement structure, mainly because of the filling effect, and would reduce the porosity of the cement composites [124,125,126,129,144,148,149,150,151]. Salemi et al. [150] reported that by addition of 2 wt.% nano-TiO_2_ to cement, the compressive strength of the cement composites increased by 27% as compared to those cement composites without TiO_2_. They found that the hydration rate of the TiO_2_-based cement composite was higher, whereas the porosity was lower than the control cement sample without nano-TiO_2_, meaning that a more compact structure with improved compressive strength would be achieved through addition of nano-TiO_2_ to the cement. Similarly, Zhang et al. [129] revealed that by addition of nano-TiO_2_, the porosity of the cementitious composites would be reduced resulting in an increase in the compressive strength of the composite. Han et al. [149] stated that by addition of SiO_2_-coated TiO_2_ to the cement, both short-term and long-term strength of the cement composite would be promoted since the SiO_2_ coating would better disperse TiO_2_ nano-particles in water because of the more negative charges on the surface of the SiO_2_-coated TiO_2._ Moreover, the SiO_2_ coating can control the size of CH crystals and also would react with them to form hydration products. Furthermore, SiO_2_ inhibits crack propagation, owing to its nanocore effect; therefore, incorporation of SiO_2_-coated TiO_2_ will significantly improve the mechanical properties of the cement composites. Figure 14 reveals the influence of SiO_2_-coated TiO_2_ on the cement composites.

Various numbers of parameters would affect the impact of nano-TiO_2_ on the compressive strength of cementitious composites including the nano-TiO_2_ dosage [130,131,152,153,154], nano-TiO_2_ size [113,134,155], and water/cement ratio [156]. As relevant researches revealed, the optimal dosage of nano-TiO_2_ directly relies on the agglomeration degree of the particles [150,157]. Regarding the impact of the size of nano-TiO_2_ on the mechanical properties of cement, Li et al. [155] explored the impact of 10 and 15 nm TiO_2_. They reported that both incorporated sizes of TiO_2_ promoted the 28-day compressive strength of the cement composite; however, the promotion obtained by 10 nm TiO_2_ was greater than that of 15 nm TiO_2_, which was related to the nucleation effect. The number of nucleating sites for 10 nm TiO_2_ was higher, while the required energy for the formation of each nucleating site was lower; thus, 10 nm TiO_2_ would lead to further enhancement in the compressive strength of the cement composite rather than 15 nm TiO_2_ [155]. Notwithstanding, self-aggregation of nano-TiO_2_ particles could conversely affect the mechanical properties of cementitious composites, which was more probable when finer nano-TiO_2_ particles were incorporated [158]. Nevertheless, some researchers reported that nano-TiO_2_ is not able to improve the mechanical properties of cementitious composites [144], and more strictly, it deteriorated the cement properties after 28 days due to the restriction of C_2_S hydration [143,157]. As reported by Li [30], addition of 10% TiO_2_ resulted in 12% reduction in the 28-day compressive strength of engineered cementitious composites (ECCs), owing to agglomeration of nano-TiO_2_ particles, which acted as flaws within the cement paste. Nevertheless, inclusion of nano-TiO_2_ would promote both the tensile and flexural strengths of ECC [30]. Therefore, more pioneering works are still required in order to clarify the influence of nano-TiO_2_ on the mechanical properties of cementitious composites.

#### 4.3.2. Flexural Strength

Similar to the compressive strength, enhancement in the flexural strength, which is an indicator of cement toughness, of TiO_2_-based cement composites has been reported by several researchers [30,122,124,125,126,129,149,151], and the following reasons have been discussed.

(1) Nucleation effect—owing to the high surface activity of nano-TiO_2_ particles, the hydration products of the cement paste would precipitate on the surface of these particles and continue to grow, forming conglomerations containing nano-particles as nucleus. This means that the nano-TiO_2_ particles dispersed in the cement matrix would promote the compactness and microstructure of the cement composites [149,159,160].

(2) Nanocore effect—due to the capability of the nano-TiO_2_ particles to deflect microcrack propagation, which is called the nanocore effect (Figure 14), these particles would have a toughening effect on the cement matrix [149].

#### 4.3.3. Shrinkage

**Figure 15:**
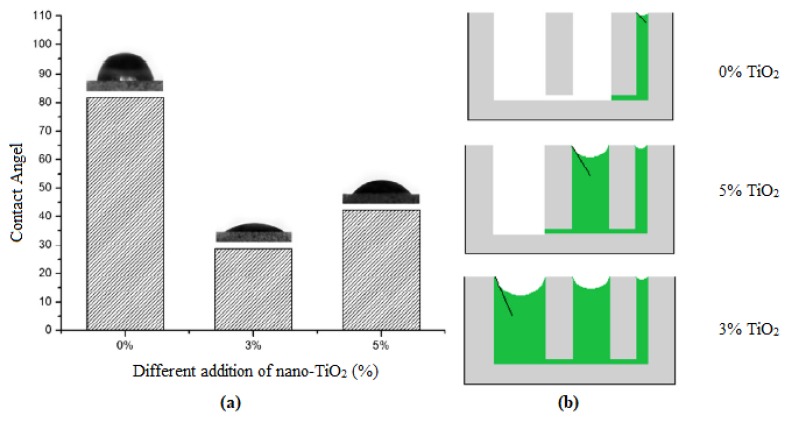
(**a**) Effect of nano-TiO_2_ addition on the cement particle contact angle. (**b**) Capillary pores filled with water before and after addition of nano-TiO_2_ [123].

Regarding the occurrence of cracks in the cementitious composites, shrinkage of the cement matrix is of significant importance, which results from the particle size distribution and characteristics of the hydration products [160,161]. Several reports revealed that the inclusion of nano-TiO_2_ results in a decrease in the microstrain of the cement matrix, meaning that the antishrinkage property of the cement matrix would be improved [162,163]. However, the opposite result has been obtained by Kurihara et al. [132]. They reported that, by incorporation of nano-TiO_2_ into the cement matrix, the shrinkage property of the cementitious composites increased, which may be due to the reduction in CH crystal size. A third pattern was also observed for the shrinkage of nano-TiO_2_-based cementitious composites, in which at the early stage, up to 6 days after preparing of the samples, shrinkage would increase, but in the range of 6 days to one month, the shrinkage of the cement composite would decrease [123,162]. The observed pattern was related to the promotion of cement hydration in the early stages and, later on, a decline in the contact angle and refinement of the pore structure of the cement matrix resulting from the addition of nano-TiO_2_ to the cement paste. A decline in the contact angle of cement particles would result in hydrophilicity of the cement paste (Figure 15) [123]. However, it is not clear yet which mechanism (i.e., reduction in the CH crystal size limiting the growth space of CH and thereby increasing the shrinkage of the cement matrix, or reducing in the contact angle of the cement particles and refining the pore structure of the cement) is the dominant factor under different conditions, meaning that more research is required in this regard.

## 5. Influential Parameters on Photocatalysis Efficiency

The mix design for photocatalytic cementitious materials, particularly in the case of white cement, is based on two fundamental pillars:

(1) The aesthetic appearance or surface finish and

(2) The strength or structural sustainability.

This means that material selection needs careful consideration in order to achieve a delicate balance between the mixture components and to guarantee the rheological behavior of the mixture [5].

**Figure 16:**
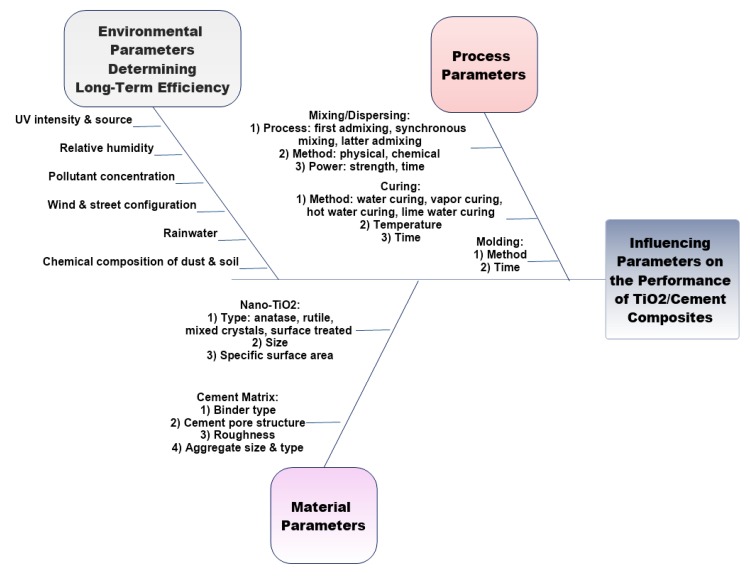
Influencing parameters affecting the performance of photocatalytic cement-based materials extracted from [137,155,166,167,168,169].

Materials selection and processing, which include mixing/dispersion, molding, and curing, are the most crucial parameters affecting performance and final properties of TiO_2_-based photocatalytic cementitious materials [31]. Figure 16 summarizes the main influential parameters on the efficiency of TiO2-based photocatalytic cementitious materials, as well as the main steps and the main methods used by researchers to incorporate and disperse nano-TiO_2_ within the cement matrix.

Regarding TiO_2_ selection, type, size, and the specific surface area of the particles are of great importance. Considering the process parameters and characteristics of the cement binder, various parameters would impact the performance of TiO_2_-based photocatalytic cement/concrete composites. Additionally, the environmental conditions are of great importance, as well since these conditions are the dominant factors that determine the long-term efficiency of the TiO_2_-based photocatalytic construction materials. The following sections will discuss the process parameters, material characteristics, and the environmental parameters in more detail.

### 5.1. Process Parameters

Mixing/dispersion methods of TiO_2_ particles in cement matrix are one of the important steps in preparation of photocatalytic cementitious composites, particularly for nano-sized TiO_2_, and they have a significant impact on the uniformity and the properties of the end-use products [6,31].

Similar to other nanoparticles, the nanosized particles and high surface energy facilitate the agglomeration of TiO_2_ nanoparticles in the cement matrix. Moreover, it is difficult to detach these agglomerates because of the high cohesion [164]. Therefore, applying an appropriate method to disperse the TiO_2_ particles within the cementitious matrix is one of the great challenges for researchers, since uniform dispersion of nano-TiO_2_ in the cement matrix during the processing of photocatalytic cementitious materials is a difficult task [165].

**Figure 17:**
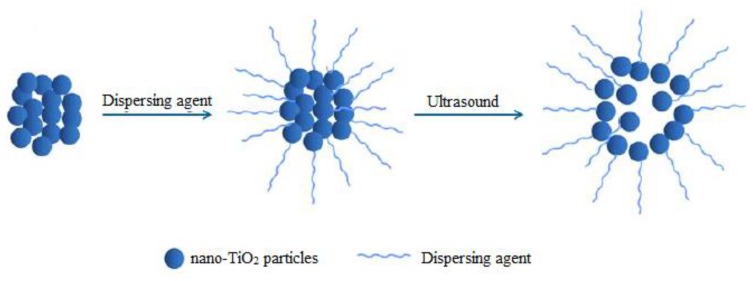
An illustration of ultrasonic dispersion of nano-TiO_2_ [31].

Considering the dispersion methods, some innovative approaches have been utilized by researchers to disperse nano-TiO_2_ in the cement matrix, including ultrasonic dispersion [125,170] and water-reducing admixtures (i.e., plasticizers and superplasticizers) [129,149]. Yang et al. [125] dispersed TiO_2_ in water using ultrasonic waves. They reported that TiO_2_ nano-particles were well dispersed in water because ultrasonic cavitation led to the formation of microjets and consequently de-aggregated the nano-TiO_2_ aggregates. Figure 17 shows a schematic diagram of the ultrasonic dispersion of nano-TiO_2_. Notwithstanding, ultrasonic techniques are expensive and would increase the total cost of preparing the photocatalysis cementitious materials based on nano-TiO_2_. As an alternative, water-reducing admixtures have been utilized to disperse nanoparticles within cement matrix. Li et al. [129] dispersed small amounts of nano-TiO_2_ in water by using water-reducing admixtures under stirring. Pérez-Nicolás et al. [171] used polycarboxylate-based superplasticizers to optimize the NO_x_ removal efficiency of cement and air lime mortars coated by titania and iron/vanadium-doped titania. They observed that inclusion of polycarboxylate-based superplasticizers would prevent agglomeration of the both nano-TiO_2_ and doped nano-TiO_2_ particles, while naphthalene sulfonate formaldehyde polycondensate would lead to the formation of large agglomerates of nanoparticles. Han et al. [149] reported that by surface treatment (e.g., SiO_2_-coated nano-TiO_2_), dispersion of nano-TiO_2_ in solution would be enhanced, since the interface of TiO_2_ modified by SiO_2_ can form Ti-O-Si bonds, resulting in more negative charge of nano-TiO_2_ particles and, hence, better dispersion of nanoparticles within the cement matrix.

On the other hand, molding methods and curing conditions can affect the porosity of TiO_2_-implemented cementitious materials and cement hydration [124,126,129,130,131,134,135,150,151,152,153,172].

### 5.2. Cement Parameters

Conducting various research towards the influential cement parameters on the efficiency of photocatalytic cement-based materials, a number of parameters has been reported in the relevant literature, which is related to the cement matrix pore structure, binder type, and cement surface roughness.

#### 5.2.1. Type of Binder

As many researchers revealed [167,173,174,175], the chemical nature of the binder would affect the photocatalytic activity of nano-TiO_2_ in construction materials. In an early attempt, Chen and Poon [114] reported a lower photocatalytic activity of ordinary Portland cement (OPC), compared to white cement, because of its metallic components. Jimenez-Relinque et al. [167] explored the effect of binder type on the photocatalytic efficiency of TiO_2_-based photocatalytic cement. They reported that the composition of the binder played an important role in the redox potential values of the aqueous phase in the pores. Also, their observations pointed out that addition of slag and fly ash would affect the photocatalytic activity negatively [167]. Their observations were further supported by findings by Andersson et al. [175]. They reported that the redox potentials of different binders were 139, –377, 106, and 131 mV for ordinary Portland cement (OPC), blast furnace slag (SC), fly ash (FAC), and calcium aluminate (CAC), respectively. This was due to the fact that in the OPC binder, most of the iron content (in the form of Fe^3+^) had potentials in the range of +100 to +200 mV [176], while SC contained a little iron, and the chemically reduced S would deactivate the electrochemically active species in high pH conditions. In another study carried out by Lee et al. [168], the photocatalytic oxidation and the binding capacity of cement containing TiO_2_ nanoparticles under exposure to NO and NO_2_ gas were explored. They found that the photocatalytic efficiencies in the removal of both gases were almost similar; however, the initial binding of NO took place at a faster rate for the pastes with higher water/cement ratios, which was due to the higher surface area. Also, in the absence of UV light, a greater binding of NO_2_ gas than NO gas to the cement matrix was observed, indicating the inherent capability of the OPC binder to bind NO_x_, particularly NO_2_.

Regarding the colored mortars containing pigments, different results have been reported. Some researchers stated that addition of iron oxides as pigment would deteriorate the photocatalytic activity of the mortar due to the interaction between the TiO_2_ and pigment [177,178], while others revealed the doping mechanism of TiO_2_ by iron oxides, thus enhancing the photocatalytic activity of the mortar [179,180]. Laplaza et al. [173] explored the photocatalytic activity of colored mortar containing iron-based pigments. They reported that the type and content of pigment would influence the photocatalytic activity of the colored mortar. The Fe/Ti ratio is a key parameter that determines the possibility of electron transfer between the conduction band of iron-based pigment and the cement mortar. This electron transfer could inhibit electron-hole recombination and higher radical formation, which upgrade the photocatalytic activity of the mortar.

#### 5.2.2. Roughness

There are a few reports regarding the effect of surface roughness on the efficiency of photocatalytic cementitious materials. As Jimenez-Relinque et al. [167] reported, for OPC, AFC, CAC, and SC binders with fine, medium, and rough surfaces, the average surface areas for medium and rough specimens were, respectively, 1.26 and 1.18 times higher than that of the fine specimen area. With the results obtained, it was observed that the NO_x_ removal efficiency increased with the roughness of the surface for all binder types. For the degradation of organic dyes, medium-roughness binders had the highest efficiency, while the rough samples had the lowest efficiency. To explain the reason for such evidence, for the self-cleaning property it is expected that all the exposed surface area has been stained with organic dye; therefore, the specimen having the most exposed area (specimen with medium roughness) is the most active one. For too rough samples, a uniform distribution of dye on the mortar surface is difficult to achieve, which might be the reason for the lowest self-cleaning efficiency of the rough specimens. Regarding NO_x_ removal, the gas is able to reach more of the specimen with a more open roughness. For medium-roughness specimens, only part of their surface area is effective for NO_x_ degradation because of their close roughness. Therefore, rough cement samples are more efficient in NO_x_ removal, followed by medium-roughness samples and then fine cement samples. In another attempt, Hot et al. [181] explored the impact of roughness on the photocatalytic activity of functional coatings. They reported that when TiO_2_ is applied to the surface, a rougher surface would allow a higher content of TiO_2_ to be incorporated. However, the highest photocatalytic activity was recorded for the surface with moderate roughness and limited content of TiO_2_, since some of the particles were inaccessible for light in too rough surfaces. Moreover, more TiO_2_ content on the too rough surfaces cannot necessarily guarantee more photocatalytic activity due to the lack of direct interaction between TiO_2_ particles, pollutants, and light.

#### 5.2.3. Cement Pore Structure

As mentioned by various researchers [31,99,167], a higher pore structure does not necessarily imply a higher photoactivity. For instance, pores larger than 1 µm (pores of air) and smaller than 0.05 µm would render a decreasing trend versus the degradation of NO_x_ and organic dyes [167]. However, Sugrañez et al. [182] reported that for the same type of cement but different sand type, cement/sand ratio, and water/cement ratio, the photocatalytic efficiency for NO_x_ removal relied on the macroporosity of the mortars. Similarly, Lucas et al. [169] observed that the photocatalytic activity would be promoted by increasing the porosity; however, there is no prevalence of pores less than 0.1 mm since small pores obstruct the diffusion of pollutants into the cementitious matrix. Ramirez et al. [183] also confirmed that a higher porosity of the substrate would enhance the efficiency of TiO_2_-coated cementitious materials for removal of toluene. Chen and Poon [114] reported a decrease in the photocatalytic activity of OPC containing TiO_2_ for NO_x_ conversion by increasing the curing time, which was attributed to the capillary pores being occupied by the hydration products and made it difficult for the pollutants and photons of light to diffuse the photocatalyst surface. The calcium-silicate-hydrate (C-S-H) gel, which is the main product of cement hydration, can form a dense coating on the surface of TiO_2_ and occupy the active sites on the surface of TiO_2_, deteriorating the photocatalytic efficiency. In recently published reports by Yang et al. [79,184], they examined the efficiency of supported TiO_2_ on quartz sand in degradation of environmental pollutants. They observed that Ti-O-Si chemical linkages were formed, and TiO_2_ particles formed uniform layers on the surface of the quartz sand. These modified TiO_2_ particles represented an enhanced binding force between aggregates and cement hydrates, leading to a photocatalytic efficiency towards degradation of NO_x_ three times higher as compared to that of conventional dispersion of TiO_2_ in mortars. In another attempt, Yang et al. [185] studied the photocatalytic efficiency of porous cement composites containing TiO_2_ for elimination of gaseous benzene. They reported that more porosity in the cement substrate was beneficial for dispersion of TiO_2_ particles, owing to the loose network pores of the needle-like hydration products, while in cement substrates with less porosity, the probability of agglomeration of TiO_2_ particles would increase. Hence, the specific surface area and the pore size distribution of the cement substrate should be optimized to achieve the highest photocatalytic efficiency. Giosuè et al. [147] studied the photocatalytic efficiency of lightweight hydraulic lime-based finishing mortar, containing expanded glass and expanded silicate as lightweight aggregates, for NO_x_ removal. Their findings revealed that using a natural hydraulic lime binder would increase the total porosity of the mortar up to 10% compared to ordinary cement binder. Moreover, the expanded glass would also add 5% more porosity to the natural hydraulic lime binder. From the results of NO_x_ removal efficiency obtained, natural hydraulic lime binder containing expanded glass revealed the highest NO_x_ conversion rate since the natural hydraulic lime binder had a higher number of large pores, while the ordinary cement binder had a higher number of small pores, meaning that in the natural hydraulic lime-based mortars, the gel pore content was lower. More of the hydration gel product present in the ordinary cement binder would result in a higher occupation of active sites of the TiO_2_.

### 5.3. Environmental Parameters Influencing the Photocatalytic Efficiency over Time

One of the most important aspect of photocatalytic functional construction materials is retaining their photocatalytic efficiency over time for both photodegredation of air pollutants and self-cleaning characteristics. There are a number of researchers exploring the long-term photocatalytic efficiency of concrete paving blocks and self-cleaning surfaces in in-site applications, in which most of them reported a decrease in the photocatalytic performance of the TiO_2_/cement composites [186,187,188,189,190]. In general, after an interval of four months, the photocatalytic efficiency of TiO_2_-based cementitious materials decreased significantly for both TiO_2_ coatings and TiO_2_ implemented in concrete bulk [191,192]. As mentioned before, by aging the photocatalytic concrete, the photocatalytic efficiency for the removal of air pollutants would decrease as a result of carbonation of the cement matrix, as well as partial deactivation of the active sites on the TiO_2_ surface, due to the adsorption of pollutants. Moreover, for TiO_2_-coated concrete components, degradation of the coating and reduction in its thickness over time would lead to more decline in the photocatalytic performance [188]. Nevertheless, as Boonen and Beeldens [186] reported, by washing the surface, the original photocatalytic efficiency would be regained, while Diamanti et al. [188] reported only 70% of the initial photocatalytic efficiency was restored after accelerated cleaning. As Witkowski et al. [187] reported, at a low UV intensity (70 w), no significant difference between the performances of the samples with different levels of cleanliness was observed; however, after cleaning the sample surfaces and applying a more intense UV source (300 w), significant NO abatement was observed.

On the other hand, there are a number of parameters that affect the photocatalytic performance of TiO_2_/cement composites for real in-site applications. Relative humidity is one of the key parameters affecting the photocatalytic activity of construction materials, which has been indicated through various in-site researches. Too high relative humidity will decrease the photocatalytic efficiency because water would be absorbed on the surface of the photocatalyst and prevent the photodegradation of the pollutants [186]. However, the initial NO_x_ concentration is also influential, as reported by [193]. When the NO concentrations were 400 and 1000 ppb, no significant effect of humidity on the NO removal was observed. Sanabria [194] reported that a relative humidity in the range of 40%–70% would not deteriorate the NO photodegradation rate. Nevertheless, Bengtsson and Castellote [195] observed a reduction in NO oxidation in a relative humidity above 40%. Moreover, since the photocatalytic performance of TiO_2_-based cementitious materials relies on direct contact between the air pollutants and the active sites of the photocatalyst, wind, street configuration, and pollution sources could impact the photocatalytic performance of cementitious materials for outdoor applications [186]. Most importantly, the UV irradiation source and intensity directly impact the ability of the photocatalysis cementitious materials to degrade the air pollutants. This could pose another challenge for retaining the photocatalytic efficiency of TiO_2_/cement composites over time, especially in countries with a low UV index [187].

To investigate the self-cleaning property of the photocatalytic concrete over time, an analysis of the self-cleaning performance of the Jubilee Church in Italy after 16 years by [189] revealed that, despite the fact that TiO_2_ was still active in the surface of the concrete, the appearance of the concrete was underperforming. As a result of this research, the author concluded that the designers need to carefully consider the way that rainwater washes the building materials in the case of using self-cleaning surfaces, since rainwater not only can cause diffuse stains on the surfaces but also can restrict the material’s performance. The latter can be seen in the case of Jubilee Church, where the abrasive effect of rainwater on the spherical surface has led to an increase in the surface roughness and, therefore, an increase in the bond between the dust particles and the concrete. The research performed by Cardellicchio [189] also revealed that the chemical composition and abrasive effect of pozzolanic dust can also jeopardize the self-cleaning property of TiO_2_/cement composites over time, which must be considered for the countries with volcanic soil and/or with frequent occurrence of desert dust.

To promote the long-term photocatalytic performance of TiO_2_/cement composites, increasing the active sites on the surface of the photocatalyst to improve the absorption of air pollutants onto the photocatalyst surface and controlling the partial deactivation of photocatalyst active sites by cement hydration products are considered as viable alternatives. As such, using a TiO_2_ carrier such as zeolite fly ash bead [196], using photocatalytic-expanded shale (PES) and photocatalytic-exposed aggregate concrete (PEAC) [191], inclusion of highly porous carbon black [197], controlling the pore structure of the cement during hydration [182], and designing macro air voids within the cement matrix [198] have been developed.

## 6. Assessment Techniques to Evaluate Photocatalytic Efficiency

**Figure 18:**
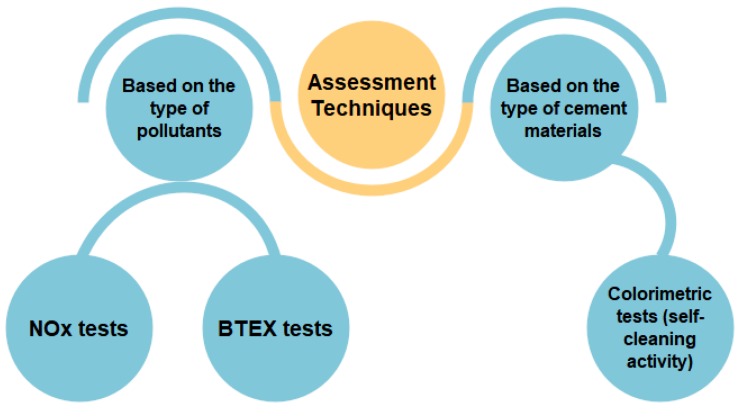
Test methods to evaluate the efficiency of photocatalytic activity in cementitious materials as reported in the literature. BTEX—benzene, toluene, ethylbenzene, and xylene.

Similar to other technologies, photocatalysis technology in cementitious materials needs to be reported in terms of qualifying and quantifying parameters in order to clarify the efficiency and workability of this innovative, functional, cement-based material. As Zhong and Haghighat [40] revealed, scientists have developed their own testing methods to assess the depolluting effect of a variety of photocatalytic materials [40], and yet there is no agreement on the most appropriate evaluation method [199]. Similarly, there is no worldwide standard to evaluate the self-cleaning property; however other available standards, which are strictly related to this property, are often used by researchers [26]. For photocatalytic cementitious materials, several test methods have been developed based on (i) type of pollutants (e.g., NO_x_, organics, etc.) and (ii) type of cement matrix (e.g., composition, physical and chemical properties, etc.). Figure 18 summarizes the main evaluation techniques for photocatalytic cementitious materials [18].

### 6.1. NO_x_ Tests

NO_x_ test series include four main categories, namely the NO_x_ flow-through test, dynamic method, static method, and Photocatalytic Innovative Coverings Applications for Depollution Assessment (PICADA) project method [18]. The NO_x_ flow-through method is a test in which the air purification performance of the photocatalytic material is assessed. This test method is in accordance to the Japanese standard JIS TR Z 0018, “Photocatalytic materials—Air purification test procedure”, in which part 1 is dedicated to the removal of nitric oxide [200]. By conducting this test method, the efficiency of the photocatalytic material would be obtained based on the measured output concentration of the sample and the concentration of NO_3_^−^ in the water in which the sample is immersed [200]. The dynamic and static test methods are commonly applied to evaluate the photocatalytic capability of inorganic materials to reduce the NO_x_ concentration [201]. All the above-mentioned test methods are capable of being adapted for VOCs as well [18]. In the European project PICADA, the photo-conversion of NO_x_ is monitored over time by means of a large testing chamber with a certain surface of photocatalytic materials at its wall [86]. In the recently published study by Jimenez-Relinque and Castellote [199], they used nitroblue tetrazolium (NBT) ink to assess the photocatalytic efficiency in high-alkaline environments, such as cementitious materials, as the first attempt to develop a monitoring method based on the NBT for the photocatalytic performance of construction materials. They reported that NBT ink is a promising alternative to conventional NO_x_ removal test methods because of its low cost, applicability to the porous, rough, and colored surfaces, less required time, in situ assessment of the photocatalytic activity, and its simplicity.

Temperature, relative humidity [79,184], and contact time (surface, flow velocity, height of the air flow over the sample, etc.) would impact the results of the tests. Generally, the efficiency of the photocatalytic process towards NO_x_ removal is promoted in the case of a longer contact time (i.e., larger surface, lower velocity, and higher turbulence), higher temperature, and lower relative humidity [18,100].

### 6.2. Benzene, Toluene, Ethylbenzene, and Xylene (BTEX) Tests

BTEX test series refer to those test methods that quantify the efficiency of the photocatalysis process in the destruction of hydrocarbon molecules including benzene, toluene, ethylbenzene, and xylene (BTEX). This test category was developed as part of the PICADA project [99,202]. The foundation of these test methods is to measure the photodegradation of organic compounds in air at ppb levels at the surface of the photocatalysis cementitious materials by means of a specially designed stirred flow reactor. The use of an actively mixed flow reactor would guarantee the uniform concentration of the reactants at the surface of the photocatalyst material [18]. This test method determines the photocatalytic activity in terms of the specific degradation rate, normalized for ultraviolet irradiation of 1000 µW cm^−2^. The obtained results would be reported as catalytic activity, expressed in (µg m^−2^ h^−1^)/ (µg m^−3^), which is equal to m h^−1^, for BTEX standard mixtures [18,40].

### 6.3. Colorimetric Tests

As part of the PICADA project, specific colorimetric tests on cement-based materials have been developed to evaluate the dye degradation and, therefore, the self-cleaning performance of photocatalytic structural materials [202]. So far, rhodamine B and methylene blue dyes have been examined [167]. Discoloration of the organic pigment, usually rhodamine B, on the TiO_2_ photocatalyst in the cement matrix is considered as evidence for photocatalytic activity (TiO_2_-sensitised photoreaction) [113]. Nevertheless, the rhodamine B test method is not applicable for porous, rough, and colored materials since, in the case of porous/rough surfaces, uniform spreading of the dye is impossible, and in the case of red-colored materials, the red color of the dye is not altered [199]. Jimenez-Relinque and Casstellote [203] suggested the application of a terephthalic acid fluorescence probe to quantitatively assess the generation rate of hydroxyl radicals, and thus the photocatalytic activity, which is a time-saving method with high accuracy. Semiquantitative methods, including utilization of reduction dyes such as resazurin (Rz) with a sacrificial electron donor, were also explored, in which their main merits were low cost, simplicity, and their applicability to the colored materials [204,205].

Evidently, the abovementioned test methods reveal the necessity of developing accurate and suitable assessment techniques that could be adapted for construction materials. The available standard methods are not capable of evaluating the photocatalytic performance of highly porous materials and/or cement, in particular for the photodegredation of NOx by cementitious materials, since the surface conditions of cementitious materials are not considered in such standard methods [206]. Likewise, a similar inaccuracy could be observed for colorimetric tests for the evaluation of the self-cleaning property in cementitious materials, which was mentioned before. For instance, some of the standard methods to test for the performance of air purification of the semiconductor photocatalysts are inapplicable for porous materials because of the high flow rates. Similarly, measurements of CO_2_ in the test method evaluating the removal of acetaldehyde is not straightforward for cementitious materials because there is high adsorption and reaction capabilities of cement with CO_2_ [199,206]. Thus, such differences in the experimental and material conditions stated in the standard methods and in the reported researches, including the irradiation source and intensity, surface roughness, cement pore structure, relative humidity, temperature, gas flow rate, initial concentration of the contaminants, and the sample size, will make it difficult to compare the obtained experimental results in order to investigate the photocatalytic efficiency and workability of a particular structural material [195]. Furthermore, incorrect assumption of the conventional test methods for removal of air pollutants (i.e., a given photocatalyst would deactivate all the pollutants equally) will lead to biased results with respect to the photocatalytic efficiency of a specific given material [199]. Ultimately, most of these standard test methods do use expensive laboratory equipment and are time-consuming.

## 7. Challenges and Future Prospects

Development of functional structural materials with superior photocatalytic activities is of great importance in terms of socioeconomic impacts and maintaining the environment, which undoubtedly represents significant technological and design challenges. Promotion of visible-light-responsive photocatalysts, leading to the effective utilization of sunlight, would be a key technological achievement in the field of photocatalysis structural materials [6,21]. As mentioned before, doping of TiO_2_ to decrease its band gap, allowing the activation of the photocatalytic process by visible light, is an active area of research [207].

Additionally, to broaden the practical applications of photocatalysis building materials, their efficiency and workability must be enhanced. In this regard, one of the promising alternatives is to improve the specific surface area of the photocatalysts [40,149]. Indeed, long-term efficiency of photocatalytic structural materials still remains as a challenge, requiring more pioneering works to (1) reduce electron-hole recombination, (2) increase the active sites on the surface of the photocatalyst, (3) control the dispersion of TiO_2_ within the cement matrix in order to achieve the optimize cluster size and porosity and to ensure maximum photocatalytic activity for both large dyes and small gaseous molecules, (4) introduce more efficient photocatalysts by coupling anatase and rutile phases, and (5) improve the cement pore structure by controlling cement hydration. Besides, further research is still required to adequately determine the decomposition performance of air contaminants and the durability of the photocatalyst itself. More importantly, full investigation of the generated by-products during the photocatalytic reaction, and their possible adverse health impacts, need careful study [21,40].

On the other hand, the impacts of photocatalyst addition on the cement composite microstructure and the long-term durability of concrete structures is another active area of research. Undoubtedly, the energy consumption would determine the future prospects of photocatalysis building materials. Moreover, coupling photocatalysis cement technology with other newborn cement technologies is a crucial part of commercialization. For instance, one of the active areas of research in the field of 3D structural printing is to introduce functional cementitious materials to the printing process and to optimize the process parameters based on the incorporated multifunctional cementitious materials [208]. Investigating the efficiency of TiO_2_ addition to other sustainable, environmentally friendly cementitious materials such as magnesium phosphate cement [209], cementitious materials based on the carbonation of fly ash, slags, and so on [210,211] could be considered as another environmental remediation for construction industry. Undoubtedly, by eliminating the present challenges, commercialization of such multifunctional structural materials would not be unattainable in near future.

## 8. Conclusions

Recent developments in the field of TiO_2_-based cementitious materials have been reviewed comprehensively. The fundamental photocatalytic oxidation process and the so-called semiconductor photocatalyst materials were discussed. Moreover, the mechanisms of self-cleaning, self-disinfecting, and depolluting effects of photocatalysis materials were described in detail. Afterwards, photocatalysis technology in the cementitious materials, relevant photochemical reactions, a critical review of the conducted research in this field, the properties of photocatalytic building materials in both fresh and hardened states, as well as assessment techniques were represented.

Apparently, the environmentally friendly technology of functional cementitious materials, with the capabilities to restore the aesthetic appearance of civil engineering structures and also to purify the air, is of great interest, especially in urban areas where the level of air pollutants has reached to concerning levels. Moreover, since civil engineering structures are a country’s largest economic investment, prolonging the aesthetic durability of these structures is of great importance. In this regard, photocatalysis cementitious materials are the superior choice to reduce the costs associated with the repair and maintenance of building facades. However, to accelerate the progress of commercialization of photocatalytic structural materials, conducting more pioneering works in order to resolve the existing technological challenges is necessary. The foremost hurdles include the development of visible-light-activating photocatalysts, improving the efficiency of photocatalysts for absorption of air pollutants, preserving the long-term efficiency of photocatalytic activity, and, more importantly, minimizing the formation of harmful by-products during photochemical reactions.
